# Gaps in HIV testing among people with presumptive TB in Mozambique: a 3-year retrospective cohort study

**DOI:** 10.1186/s12879-025-11545-7

**Published:** 2025-09-26

**Authors:** Guita Amane, Paula Simbine, Pereira Zindoga, Algy Abdula, Miguelhete Lisboa

**Affiliations:** 1 Ministério da Saúde, Direcção Nacional de Saúde Pública, Programa Nacional de Controlo de ITS HIV/SIDA, Maputo, Mozambique; 2 US Agency for International Development, Integrated Health Office, P.O. Box 783, Marginal Avenue, 5467, Maputo, Mozambique; 3 Development Aid from People to People (ADPP), Mozambique Local TB Response, USAID TB project, Matola, Mozambique

**Keywords:** HIV testing, Presumptive tuberculosis, HIV/TB integration, Community-based TB interventions, Mozambique

## Abstract

**Background:**

HIV testing among people with presumptive tuberculosis (PP-TB) represents a critical entry point for HIV diagnosis and care, especially in high-burden settings like Mozambique. However, systematic testing in this population remains suboptimal. This study assessed gaps in HIV testing across the TB care cascade.

**Methods:**

We conducted a retrospective cohort study using programmatic data from clients screened for TB through community-based TB interventions in four high TB burden provinces from 2021 to 2023. Data was extracted from the registry books and triangulated with an electronic reporting system used by the national TB program. Descriptive analysis was conducted to identify drop-offs in HIV testing among PP-TB, particularly at three key stages: community screening, facility-based TB evaluation, and among confirmed TB clients.

**Results:**

Among the 4,607,257 clients screened, 52% were female, and 62% were aged 15 years and older. Of those screened, 9% (415,654) were identified as PP-TB from whom 85% (351,255) were referred to health facilities, 97% (341,049) successfully completed referrals, and 96% (326,664) were further evaluated for TB. Of those evaluated, 24% (77,584) were diagnosed with TB and 85% were notified and initiated anti-TB treatment. Three levels of gaps in HIV testing were identified: (i) HIV testing omission, no evidence of concurrent HIV testing was documented at community level – community TB lay staff not allowed to perform HIV testing; (ii) HIV testing gap among TB-negative clients, 76% were not tested for HIV, despite their presumptive TB status; (iii) HIV testing deficiency among confirmed TB clients, 14% of these confirmed TB remained with unknown HIV status. Combining these sequential testing failures, the estimated overall gap in HIV testing among PP-TB reached 84%.

**Conclusion:**

HIV testing integration into TB care cascade remains limited in Mozambique, with substantial missed opportunities at the community level, among TB-negative clients, and even among confirmed TB clients. Strengthening HIV testing as a systematic component of TB screening, especially in outreach context, could enhance early diagnosis, linkage to care, and co-infection management. These findings call for urgent policy and operational adjustments to close the gaps in HIV testing, particularly within the community-based TB services.

## Background

Mozambique is listed among the global high-burden countries for tuberculosis (TB) and HIV-associated TB, with an incidence rate of 361 cases per 100,000 population, a TB and HIV co-infection rate of 25%, and 4,300 deaths from HIV-associated TB in 2022 [1]. This high mortality rate is largely attributed to delays in diagnosing both HIV and TB and the lack of timely and effective treatment [2].

The World Health Organization (WHO) recommends the integration of TB and HIV control, including integrated community-based multi-disease testing strategies to reduce stigma by normalizing HIV and TB testing [3]. The WHO has been issuing policy statements and guidelines on TB and HIV collaborative activities and differentiated HIV testing services (dHTS) for several years. In 2012, WHO updated its recommendations, advocating that routine dHTS should be provided not only to patients diagnosed with TB but also to people with presumptive TB, whether at community or health facility levels [4]. These recommendations were reinforced in 2019 and updated in the 2024 WHO consolidated guidelines on dHTS [5, 6], based on studies from Sub-Saharan African countries that show a high HIV prevalence among people with presumptive TB, ranging from 27 to 64% [7–11].

Despite these guidelines being in place for over a decade, the provision of TB and HIV integrated services in Mozambique has primarily been offered at facility level and only to patients diagnosed with TB and not to PP-TB. When HTS are offered at facility level, testing volumes for PP-TB remain below expectations, despite this being a high-yield HIV case-finding strategy [12].

At community level, HTS are not offered to PP- TB at all. This is partly because existing community-based TB and HIV services are not integrated, making it more costly and complicated for people to be screened for both TB and HIV [13].

Therefore, while HIV testing coverage among patients diagnosed with TB at health facilities generally exceeds 95% with high testing positivity, there remains a significant opportunity gap in identifying and HIV testing PP-TB at both community and facility levels [12, 14]. However, there is limited information on the extent of opportunity gaps in HIV testing and context-based factors throughout the TB screening and testing cascade from the community to the health facilities. This study was conducted to assess gaps in HIV testing among PP-TB from the community to the facility-based TB care cascade.

## Methods

### Study design

We conducted a retrospective cohort study using routine data from clients reached and screened for TB through community-based TB interventions in four high TB burden provinces (Nampula, Sofala, Tete, and Zambézia) over a 3-year period (2021–2023).

### Population, sample, and inclusion or exclusion criteria

We purposively retrieved data on all PP-TB who were reached through community-based TB interventions supported by the Mozambique Local TB Response (LTBR) project in Nampula, Sofala, Tete, and Zambézia provinces. PP-TB reached through prison- or facility-based interventions, as well as those reached by unknown modalities, were excluded from the study.

### Study setting

Mozambique has a population of 32,419,747, with approximately 57% residing in the four high TB burden provinces: Nampula (6.6 million), Zambézia (6 million), Tete (3.2 million), and Sofala (2.6 million) [15].

**Fig. 1:**
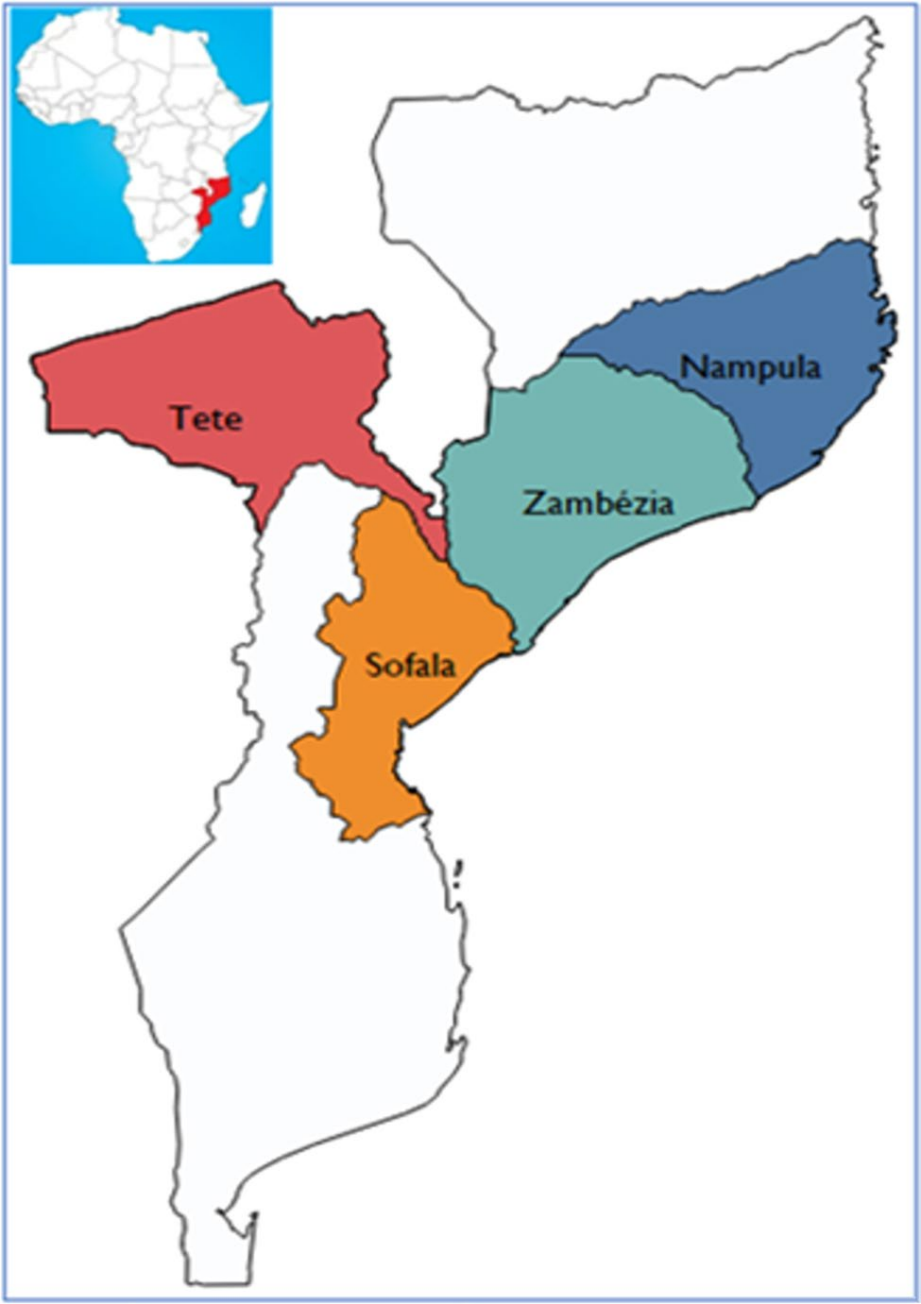
USAID-supported provinces through Mozambique Local TB Response (LTBR)

This study was conducted in these four provinces – (Fig. 1) covering 50 districts. About 54% (62,934 out of 116,817) of all TB cases in the country notified in 2023 were from these 4 provinces [12]. The LTBR project, supported by the United States Agency for International Development (USAID) and implemented by Development Aid from People to People (ADPP) in collaboration with the Ministry of Health (MoH), aims to enhance local and community-based TB services in these four provinces. The project’s goals include improving the quality of patient-centered TB care, increasing the diagnosis of both drug-sensitive and drug-resistant TB, and boosting treatment success rates.

### Data sources and variables

Data was extracted from the registry books and triangulated with an electronic reporting system (District Health Information Software version 2, DHIS2) used by the national TB program. The study variables included the following aggregated numbers of clients who were reached through community-based TB interventions, number of clients screened for TB, number of clients who were referred to health facilities for further TB investigation, number of clients who underwent clinical and laboratorial TB evaluations, number of clients who were clinically or bacteriologically diagnosed with all forms of TB, number of clients who were tested for HIV alongside TB care cascade.

### Data analysis

We conducted a descriptive care cascade analysis (proportional drop-offs) to quantify drop-offs in HIV testing at each stage of the TB care cascade among PP-TB who were reached through community-based TB interventions. We produced visual care cascade charts using MS-Excel to give a clear snapshot of where PP-TB were not tested for HIV, particularly at three key stages: community screening, facility-based TB evaluation, and among confirmed TB clients.

## Results

### Client characteristics

**Fig. 2:**
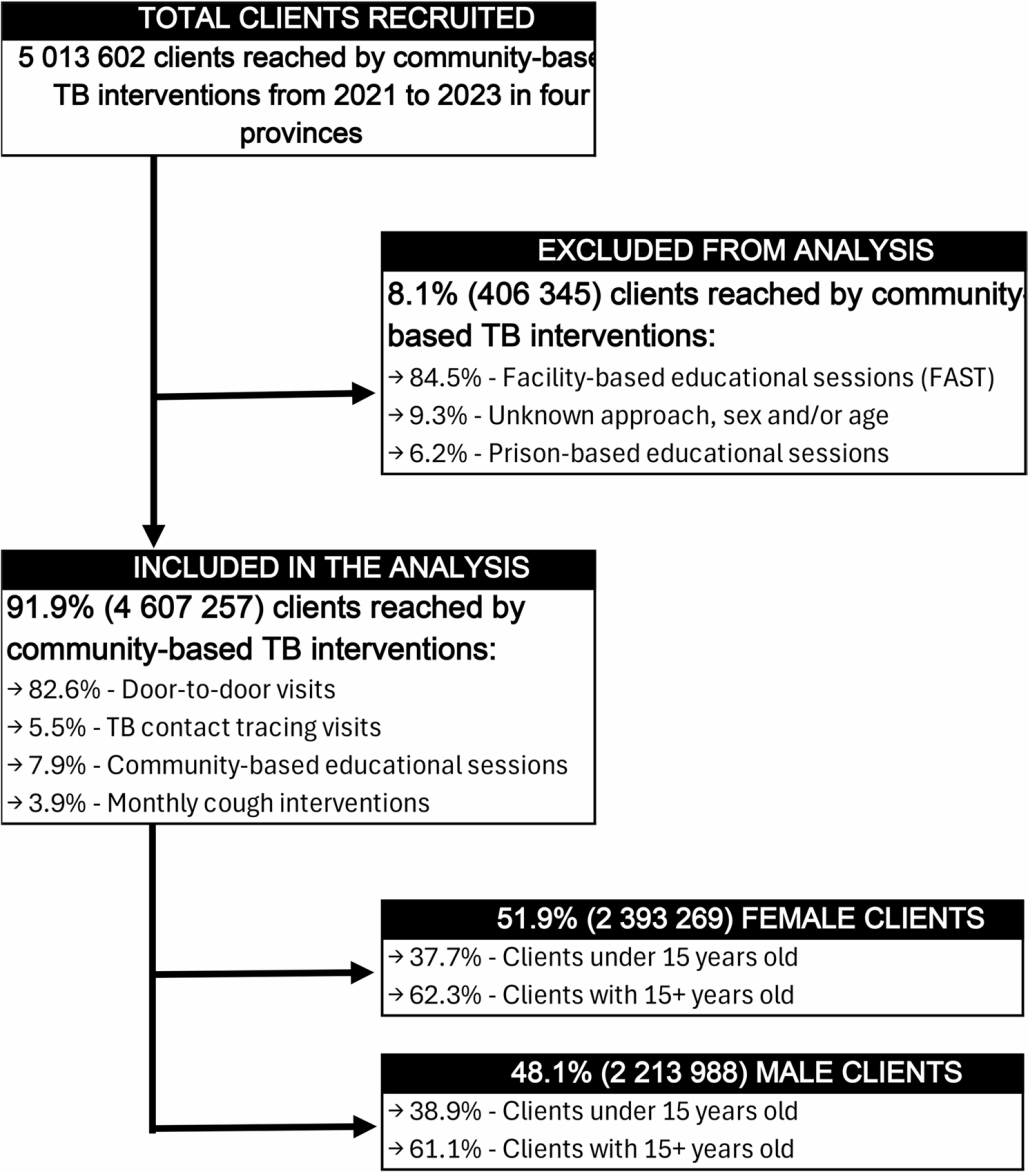
Schematic representation of study population and selection procedure

Between January 2021 and December 2023, a total of 5,013,602 clients were reached through community-based TB interventions. Out of this total, 91.9% (4,607,257) were included in the data analysis as they were reached out exclusively through community-based TB interventions and had complete information. The remaining clients were excluded due to missing or incomplete information (9.3%), reached out through non-community settings such as prisons (6.2%) or reached out through health facility waiting rooms (9.3%) - Finding TB cases Actively, Separating Safely, and Treating Effectively (FAST) strategy (Fig. 2).

Among the 4,607,257 clients included in the analysis, approximately 52% (2,393,269) were female, and 61.8% (2,845,435) were aged 15 years and older. Most of these clients were reached out through community door-to-door visits (82.6%, or 3,805,188), followed by public gathering places (7.9%, or 366,195), community TB contact tracing visits (5.5%, or 254,314), and monthly cough interventions (3.9%, or 181,560). Most clients were reached out in Zambézia (32.6%), Nampula (30.8%), and Sofala (27.3%), which together accounted for 90.7% of the total.

### TB care cascade

#### Community-level TB screening and referral

Out of 4,607,257 clients who were reached out and screened for TB, 9% (415,654) were identified to have signs and symptoms of TB, therefore, considered PP-TB. Among these, 84.5% (351,255) were referred to health facilities, which means that 15.5% (64,399) of PP-TB were lost due to either refusal to provide a sputum sample or refusal to accept the referral document to present themselves at a health facility. Approximately 2.9% (10,206) and 4.2% (14,385) of PP-TB were lost from the community referral to the facility and then to TB evaluation, respectively. These losses can be attributed to the low perception of TB risk, the long distances required to reach a facility, long waiting times, and the behavior of healthcare providers towards clients referred to health facilities by community TB workers.

#### Facility-level TB evaluation and diagnosis

Of those PP-TB who referred to health facilities, 97% (341,049) successfully completed referrals, and 95.8% of them were further evaluated for TB using laboratory technologies or clinical assessment, including X-rays. Among those PP-TB who were further evaluated for TB, 23.7% (77,584) were confirmed to have TB, of whom 85.4% (66,272) were notified and initiated anti-TB treatment.

**Fig. 3:**
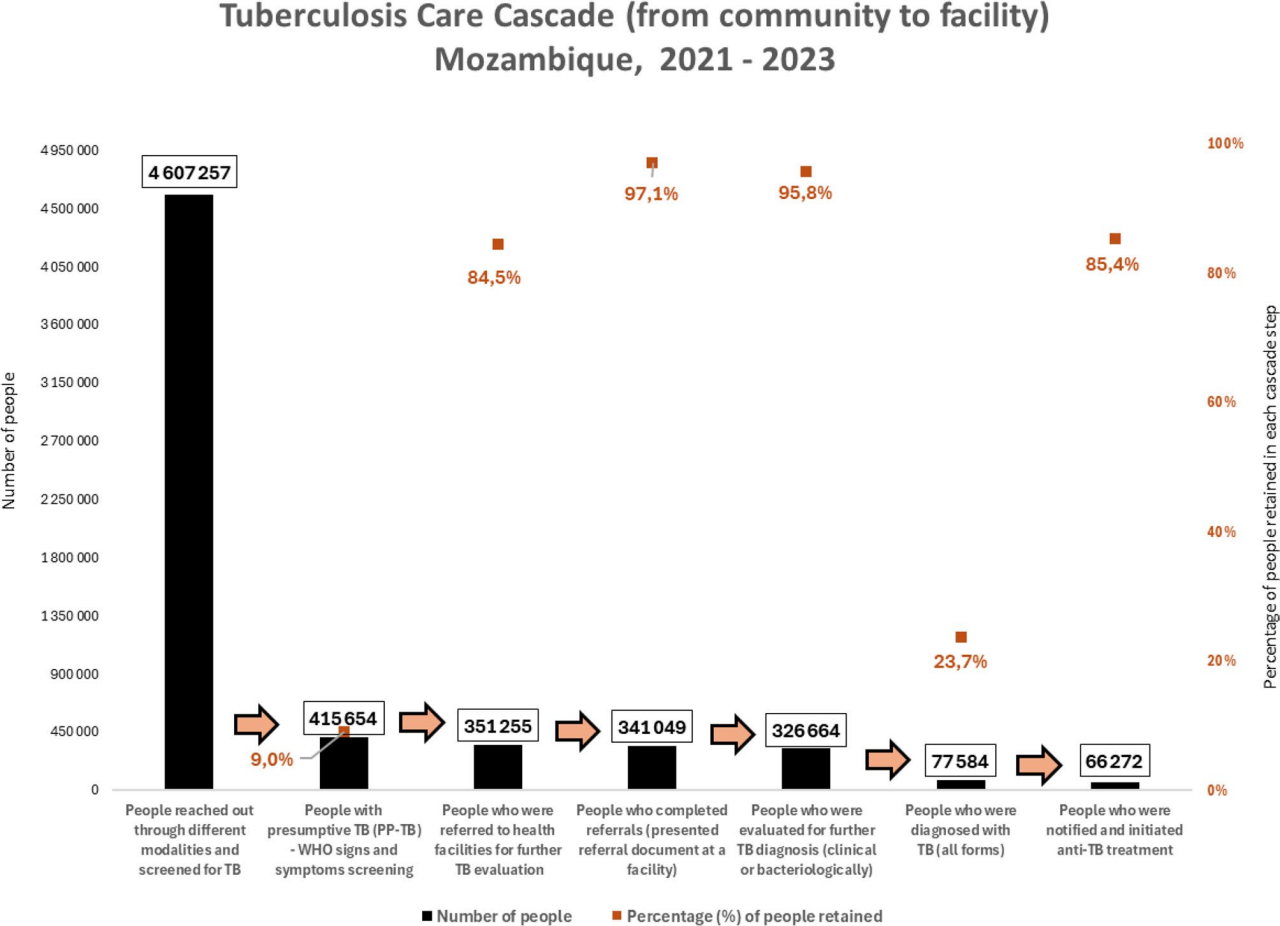
Overall outcomes of the TB care cascade (from community to facility)

Approximately 14.6% (11,312) of the PP-TB patients were lost to follow-up between TB diagnosis and treatment initiation, largely due to the unavailability of same-day diagnostic results, as most sputum smear examination results are delivered the following days by healthcare workers at facility level or by community lay TB workers. Consequently, tracing these patients in a timely manner for enrollment into TB care and treatment can be challenging, particularly for those who were initially reached through public gatherings and monthly ‘cough day’ interventions (Fig. 3).

### HIV testing among PP-TB

#### Community-level HIV testing omission

Alongside the community-based TB screening and referral, although 9% (415,654) of screened clients were identified to be PP-TB, gaps in HIV testing emerged at multiple critical junctures along the diagnostic and referral continuum, as no evidence of concurrent HIV testing was documented at this stage. This omission represents the first major gap in HIV testing, wherein all PP-TB initially identified at the community level likely missed the opportunity to know their HIV status.

#### Facility-level HIV testing gap among TB-negative clients

**Fig. 4:**
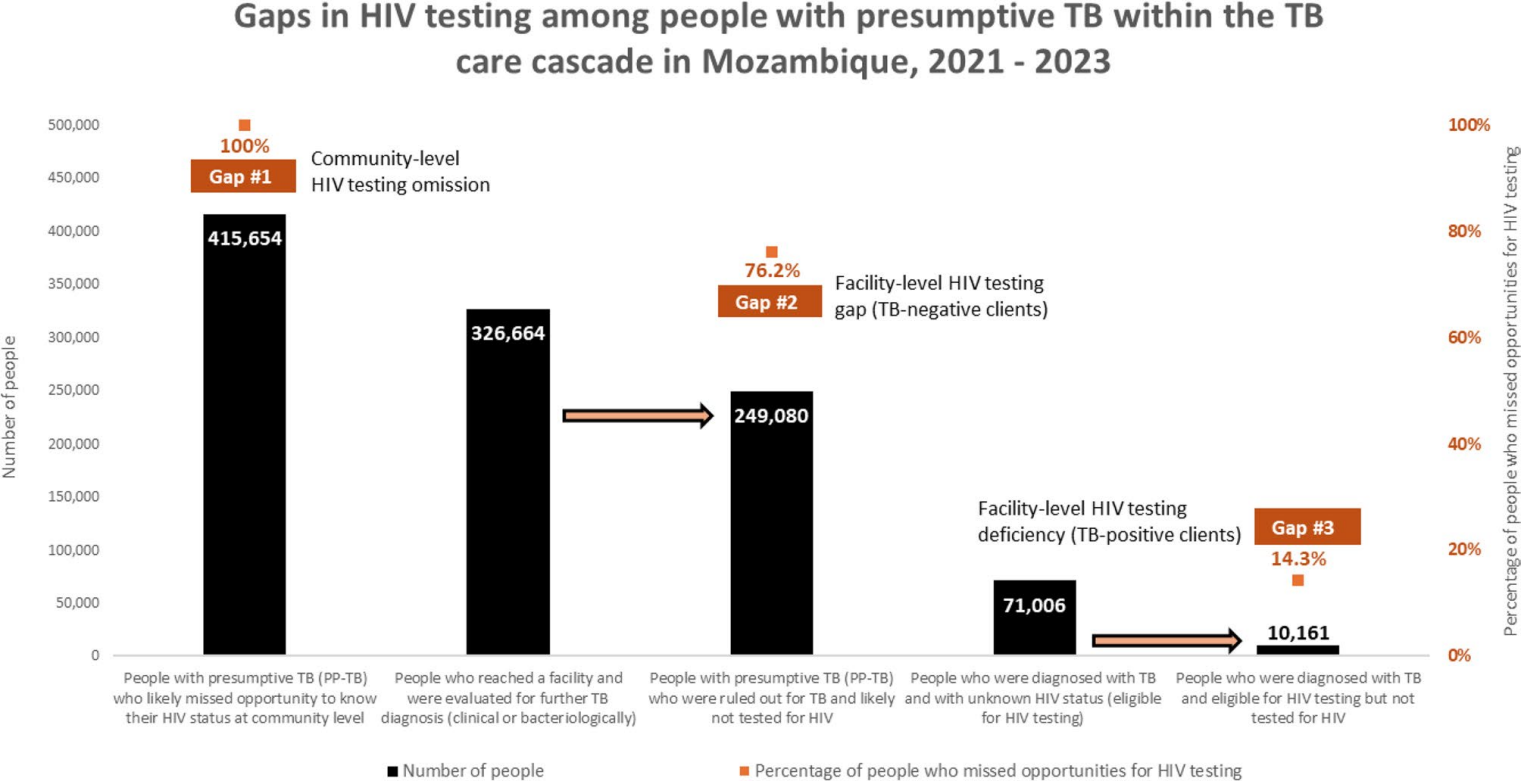
Gaps in HIV testing among people with presumptive TB

Of the 415,654 PP-TB, 85% (351,255) were referred to health facilities, with a successful referral completion rate of 97% (341,049). Of these, 96% (326,664) underwent clinical and bacteriological TB evaluation. However, among those who were subsequently ruled out for TB, 76.2% were not tested for HIV, despite their presumptive TB status, which should have flagged them as high priority for testing. This is the second major gap in HIV testing and represents a lost opportunity to identify individuals with HIV who may be present with TB-like symptoms but do not meet diagnostic criteria for TB - Fig. 4.

#### Facility-level HIV testing deficiency among confirmed TB-positive clients

Among the 326,664 PP-TB clients evaluated, 23.7% (77,584) were diagnosed with TB, and the majority (85%) were promptly notified and initiated treatment. Among 71,006 PP-TB eligible for HIV testing (6,578 out of 77,584 were already living with HIV), 14.3% (10,161) remained with unknown HIV status as there was not any evidence of HIV testing. This is the third major gap in HIV testing, signaling a critical gap in co-infection management and missed linkage to HIV care - Fig. 4.

#### Overall missed HIV testing opportunities

Combining these sequential testing failures, the estimated overall gap in HIV testing among PP-TB reached 83.7% (348,231/415,654). It means that only a total of 67,423 out of 415,654 PP-TB were excluded from the estimation of the overall gap in HIV testing because 6,578 (1.6%) were not eligible for HIV testing (known to be already living with HIV), and 60,845 (14.9%) were tested for HIV at facility level after being diagnosed with TB disease. That said, all remaining PP-TB, such as the presumptive patients who presented to health facility but were not evaluated for TB and were also not tested for HIV or patients tested negative for TB and were not tested for HIV were considered.

This cascade of missed opportunities suggests systemic weaknesses in HIV integration within TB screening protocols, particularly within community outreach and among TB-negative clients, who may still be at high risk of HIV infection.

## Discussion

The findings of this study demonstrate that while HIV testing among PP-TB is highly recommended [4–6], Mozambique still lags in the integration of HIV and TB interventions at both, community and facility levels. HIV testing coverage was strikingly limited, despite the clear intersection between HIV and TB risk, and gaps in HIV testing emerged at multiple critical junctures along the screening, referral and diagnostic continuum. The gaps related to community-level HIV testing omission among PP-TB are likely due to lack of national policy (guideline or strategy) on HIV and TB integration at community level. As a result, either community-based lay TB workers are not allowed to perform HIV testing or community-based lay HIV counselors to perform TB screening and collection of sputum samples.

Apart from the challenge of effective integration of HIV and TB interventions, the gaps in HIV testing are likely fueled by lack of knowledge and/or skills to perform HIV testing, and lack of technical assistance of the healthcare providers at facility level. This observation is in alignment with lessons learned and documented by Uwimana et al. [16] where a combination of upskilled community lay health workers and enhanced supervision and technical assistance were potential to bridge gaps in service delivery, increasing TB screening and HIV testing coverage, and ultimately HIV/TB case finding. In addition, integrating HIV and TB interventions at the community level might also reduce the proportion of PP-TB who missed opportunity for HIV testing related to the referral processes from the community to health facilities, which was estimated at 26.6% (110,605) in this study.

The lack of integration of HIV and TB interventions at community level also leads to the underutilization of the existing community lay health workforce. Community lay TB workers, given their roles, are well-suited to educate people about HIV, perform HIV testing for those with unknown HIV status, and refer individuals for HIV prevention combination or care and treatment services. Similarly, community lay HIV workers, such as HIV counselors and case managers, are ideally positioned to educate patients about TB risks and symptoms, screen for TB signs and symptoms, refer them for further TB evaluation, and support psychosocially those undergoing TB treatment or preventive TB therapy.

Based on lessons learned from South Africa [16, 17], community-based integrated TB and HIV interventions, particularly in settings with high prevalence of both HIV and TB and limited health financing resources, such as Mozambique, are likely the most effective approaches for accelerating the implementation of collaborative HIV and TB interventions. By integrating these roles and enhancing vigilance, as observed in South Africa [16], the detection of both HIV and TB cases within these high-risk populations could be significantly improved. Additionally, providing HIV and TB services through a single community lay health worker, rather than multiple lay workers, would result in more comprehensive care, reducing the need for multiple visits and lowering associated costs [16, 18].

We also observed that at the facility level, almost all PP-TB who were ruled out for TB were not tested for HIV. Regrettably, about 64% of the health facilities still use sputum smear microscopy as the primary diagnostic tool to rule out PP-TB [12]. However, using sputum smear microscopy method does not guarantee that the PP-TB are 100% TB-free due to its own limitations in both sensitivity and specificity for TB diagnosis [18, 19]. Additionally, evidence from many Sub-Saharan African countries such as Kenya, Ghana, Uganda, Ethiopia, Guinea Bissau demonstrated that HIV prevalence among PP-TB who were ruled out for TB was high and even higher than HIV prevalence among people diagnosed with TB [7–11]. A study conducted in Mozambique also demonstrated a high HIV positivity rate among people with presumptive TB with unknown HIV status, about 12% among adults and 3% among children [20]. Thus, as recommended by Frasca et al. [21], it is critical to perform HIV testing to PP-TB at the time of diagnosis of TB, even if the sputum smear microscopy result is negative.

Finally, we observed a discrepancy between national data, which report that only 1% of TB patients were not tested for HIV, and our study findings (14.3%). This divergence highlights important operational gaps in HIV service delivery at the facility level and likely reflects limitations in the continuum of care, particularly at the interface between community-based TB case finding and facility-based HIV testing. Patients diagnosed through community outreach strategies, such as public gatherings or “cough day” interventions, as noted by WHO [22], may not be systematically or promptly linked to facility-based services offering provider-initiated HIV testing and counselling, thus increasing the likelihood of missed testing opportunities.

In addition, the unavailability of same-day sputum smear examination results may contribute to the lack of prompt provision of integrated TB/HIV services, particularly during the patient’s initial visit. Moreover, our study relied on aggregated data, which can obscure individual-level dynamics and re-engagement patterns. For instance, individuals who initially missed HIV testing may have been tested later during follow-up visits or at a different health facility. Such delayed engagements, as observed by Suthar et al. [23], may not be adequately captured in routine health information systems.

The main limitation of the study design is the use of retrospective data solely from community-based TB interventions, where lay health workers are neither trained nor equipped to perform HIV testing. Therefore, the number of PP-TB who were likely lost for HIV testing might not accurately reflect the true figures, as some clients might already know their HIV status and may even be enrolled in an HIV care and treatment program. In addition, we used aggregated data, which may not be sufficient to understand individual-level or specific risk factors associated with gaps in HIV testing within the screening and testing cascade nor to perform detailed subgroup analysis. However, the fact that at community level there is not at all an integration between HIV and TB screening and testing services, technical guidance, monitoring and evaluation tools, we believe it may clearly result in a significant number of PP-TB who are not tested for HIV. In addition, at least for those PP-TB that have successfully completed referral and were further evaluated for TB at facility level, we managed to identify who were eligible or not for HIV testing and we took out from the denominator. By saying it is not eligible we mean any PP-TB who were already living with HIV or had valid evidence with an updated HIV testing.

## Conclusion

The findings of this study show a limited HIV testing integration into TB care cascade in Mozambique, with substantial missed opportunities at the community level, among TB-negative clients and even among confirmed TB clients, at facility level. Strengthening HIV testing as a systematic component of TB screening, especially in outreach context, could enhance early diagnosis, linkage to care, and co-infection management for both HIV and TB infections. These findings call for urgent policy and operational adjustments to close the gaps in HIV testing, particularly within the community-based TB services.

## Data Availability

The data that support the findings of this study are available from Mozambique Ministry of Health, but restrictions apply to the availability of these data, which were used under license for the current study, and so are not publicly available. Data are however available from the authors upon reasonable request and with permission of the Mozambique Ministry of Health.

## Abbreviations

AA: Algy Abdula
dHTS: differentiated HIV Testing Services
GA: Guita Amane
HIV: Human Immunodeficiency Virus
LTBR: Mozambique Local TB Response
ML: Miguelhete Lisboa
MoH: Ministry of Health
PP-TB: People with Presumptive Tuberculosis
PS: Paula Simbine
PZ: Pereira Zindoga
TB: Tuberculosis
USAID: United States Agency for International Development
WHO: World Health Organization
